# Presence of problematic and disordered gambling in older age and validation of the South Oaks Gambling Scale

**DOI:** 10.1371/journal.pone.0233222

**Published:** 2020-05-19

**Authors:** Roser Granero, Susana Jiménez-Murcia, Fernando Fernández-Aranda, Amparo del Pino-Gutiérrez, Teresa Mena-Moreno, Gemma Mestre-Bach, Mónica Gómez-Peña, Laura Moragas, Neus Aymamí, Isabelle Giroux, Marie Grall-Bronnec, Anne Sauvaget, Ester Codina, Cristina Vintró-Alcaraz, María Lozano-Madrid, Marco Camozzi, Zaida Agüera, Jéssica Sánchez-González, Gemma Casalé-Salayet, Isabel Sánchez, Hibai López-González, Eduardo Valenciano-Mendoza, Bernat Mora, Isabel Baenas, José M. Menchón

**Affiliations:** 1 Ciber Fisiopatología Obesidad y Nutrición (CIBERObn), Instituto de Salud Carlos III. Madrid, Spain; 2 Department of Psychobiology and Methodology, Autonomous University of Barcelona, Barcelona, Spain; 3 Department of Psychiatry, University Hospital of Bellvitge-IDIBELL, Barcelona, Spain; 4 Department of Clinical Sciences, School of Medicine and Health Sciences, University of Barcelona, Barcelona, Spain; 5 Department of Public Health, Mental Health and Perinatal Nursing, School of Nursing, University of Barcelona, Barcelona, Spain; 6 Centre d'Excellence pour la Prévention et le Traitement du Jeu, Faculté de Sciencies Sociales, Université Laval, Pavillon Félix-Antoine-Savard, Quebec, QC, Canada; 7 INSERM, SPHERE U1246, University of Nantes, Nantes, France; 8 CHU Nantes, Movement—Interactions—Performance, University of Nantes, MIP, EA 4334, Nantes, France; 9 Ciber Salut Mental (CIBERSam), Instituto de Salud Carlos III. Madrid, Spain; eCampus University, ITALY

## Abstract

The use of instruments originally developed for measuring gambling activity in younger populations may not be appropriate in older age individuals. The aim of this study was to examine the presence of problematic and disordered gambling in seniors aged 50 or over, and study the reliability and validity properties of the SOGS (a screening measure to identify gambling related problems). Two independent samples were recruited: a clinical group of *n* = 47 patients seeking treatment at a Pathological Gambling Outpatient Unit, and a population-based group of *n* = 361 participants recruited from the same geographical area. Confirmatory factor analysis verified the bifactor structure for the SOGS with two correlated underlying dimensions [measuring the impact of gambling on the self primarily (Cronbach’s alpha α = 0.87) or on both the self and others also (α = 0.82)], and a global dimension of gambling severity (also with excellent internal consistency, α = 0.90). The SOG obtained excellent accuracy/validity for identifying gambling severity based on the DSM-5 criteria (area under the ROC curve AUC = 0.97 for discriminating disordered gambling and AUC = 0.91 for discriminating problem gambling), and good convergent validity with external measures of gambling (Pearson’s correlation R = 0.91 with the total number of DSM-5 criteria for gambling disorder, and R = 0.55 with the debts accumulated due to gambling) and psychopathology (R = 0.50, 0.43 and 0.44 with the SCL-90R depression, anxiety and GSI scales). The optimal cutoff point for identifying gambling disorder was 4 (sensitivity Se = 92.3% and specificity Sp = 98.6%) and 2 for identifying problem gambling (Se = 78.8% and Sp = 96.7%). This study provides empirical support for the reliability and validity of the SOGS for assessing problem gambling in elders, and identifies two specific factors that could help both research and clinical decision-making, based on the severity and consequences of the gambling activity.

## Introduction

Many elders enjoy recreational gambling without suffering clinical consequences, but age-specific risk factors can make other older individuals highly vulnerable to problematic gambling, defined as a wide spectrum of harmful behaviors related to gambling activity, among which the most dysfunctional and severe form is gambling disorder (GD). In the psychiatric area, GD is defined as the persistent and recurrent need to gamble, leading to clinically significant impairment and distress [1].

Worldwide prevalence rates for this disorder have showed significant increases in recent decades among all age groups, from adolescence to old age, with lifetime rates in older population-based samples estimated to be between 0.01% to 11% [2,3]. In fact, epidemiological studies assessing the incidence rate of GD in the elderly population are scarce, and the available current data provide prevalences into a high range. While some researches indicate that incidence for GD may be twice as high in young adults compared to older adults [4], other studies have reported rates in older populations quite similar to those obtained in younger groups: into the range 0.1% to 11% for lifespan problem gambling [2] and between 1% to 2% assessing problem gambling during the last year [3]. It has also been observed that around 50% of adults aged over 60 have a history of lifetime gambling, around 70% have gambled in the last 12 months, and around 2% are into the group of high risk for problematic gambling [5].

The transition from middle-age adulthood to older age is a critical phase, with major adjustments and changes that can significantly affect gambling habits. The most relevant risk factors for GD are socio-demographical variations (e.g., retirement, financial hardship or social isolation) [6,7], neurological vulnerabilities in the mechanisms associated with behavioral regulation and diminished executive functioning (e.g., due to the aging process, substance-related impairments or other physical disorders such as Parkinson’s) [8–10], and the poor physical and psychological health typical of aging, involving chronic medical conditions, limited mobility, anxiety or depression) [11–15]. The universalization of the new digital systems even among elders (particularly Internet, whose expansion increases the opportunities for accessing multiple online gambling activities) [16] constitutes another relevant risk factor for problematic and disordered gambling [17,18].

The assessment of gambling in older age is currently based on screening and diagnostic tests (many of them administered in self-report and paper-and-pencil format), whose results constitute the basis for the clinical judgments about the individual’s gambling profile and for decision-making regarding his or her therapeutic needs. However, these tools were originally developed and validated in samples of largely younger individuals ranging in age from adolescence to middle-age, and, to our knowledge, these self-report instruments have not been psychometrically evaluated to guarantee their reliability and validity for use among older age individuals either in population-based samples or in clinical settings. Therefore, it is not clear that the content of the items, their factorial structure, or the cutoff points are really appropriate to assess the specific characteristics of problem gambling in later life.

Various measurement instruments are currently used in this age group, such as the Canadian Problem Gambling Instrument (CPGI) [19] and several questionnaires based on the *Diagnostic and Statistical Manual of Mental Disorders* (DSM) [e.g., the Diagnostic Questionnaire for Pathological Gambling [20], the National Opinion Research Center DSM Screen for Gambling Problems NODS [21], the Diagnostic Interview for Gambling Severity DIGS [22] or the Lie/Bet Questionnaire for Screening Pathological Gamblers [23]].

One of the pioneering tools specifically developed for screening gambling problems was the South Oaks Gambling Screen (SOGS) [24], based on the operational definition for pathological gambling in the DSM–III edition [25] and usually administered as a self-report instrument (although it also allows other modes, including interviews conducted by others such as professional clinical and non-professional interviewers, telephone or computer). The SOGS, which is currently the most widely used instrument, includes 20 multiple-choice items originally structured in a one-single dimension (17 additional non-scoring items also allow the identification of the type of gambling, the amount of money gambled, and the presence of other relatives or close friends with problem gambling behaviors). Primarily developed to screen for pathological gamblers in clinical settings, the SOGS has been translated into a number of languages and used in a variety of countries in clinical and research settings as a screening tool for rapid and accurate identification of individuals at high risk of problematic and disordered gambling, as well as to measure the changes over time after treatments applied to reduce/control gambling behaviors. Many studies of the SOGS have obtained satisfactory results for its psychometric properties (according to the classical coefficients of internal consistency, test-retest reliability and convergent validity) [26].

Four main critiques have been postulated regarding the SOGS. The first is that its contents were not well matched to the DSM criteria for disordered gambling. The tool covers cognitive, emotional and other behaviors related to problem gambling, with items measuring lying about gambling activity, losses and debts, taking time off work, arguments with family or close friends, feeling guilty, borrowing money to gamble, and performing illegal acts to finance gambling. It has been argued that the items examining the consequences of gambling are considerably more numerous than the items specifically measuring gambling behavior [27,28]; that phenomena strongly related to the addiction model considered in the DSM-III-R are not covered in the SOGS questions (such as withdrawal and tolerance); and that borrowing money for finance gambling is overrepresented in the tool (given that 10 items are devoted to measuring this problem).

The second point of criticism is related to the questionnaire’s construct validity, since it lacks a clearly stated definition of problematic and disordered gambling. It has been postulated that the items were developed as a mixture of the DSM-III criteria plus other inductive/deductive approaches based on the scientific literature for gambling (particularly, the questions related to impaired impulse control and loss-chasing) and on professional judgment [29].

The third criticism of the SOGS refers to its scoring, which has typically taken the form of two broad measures: a dimensional score for gambling severity (generated as the sum of the 20 items), and a categorical classification of the gambling level based on a cutoff point (which discriminates between gamblers with a low risk of problems versus probable pathological gamblers). While it has been stated that the dimensional SOGS score may be a useful measure of gambling severity in both clinical and research settings (Goodie et al., 2013), a wide range of difficulties have been highlighted relating to the use of the SOGS in a categorical manner for screening the presence of possible problem gambling. The optimal cutoff point was primarily fixed at 5, but this choice has been questioned due to the absence of rigorous statistical procedures such as Receiver Operating Characteristic Curve Analyses (the 5-point threshold simply minimized false positives, false alarm rates, and balanced with false negatives among the individuals of the study) (Pepe, 2003). Since the use of this cutoff has also reported hazardous increases in the likelihood of false negative errors in population-based samples, other lower thresholds have been proposed to maximize the accuracy and to balance sensitivity and specificity [30,31]. However, lowering the cutoff for the SOGS has produced significantly higher prevalence rates in the general populations than those estimated with other diagnostic tools (such as those based on the DSM criteria). Whatever the case, the concerns about the excessive rates of false alarms and false discoveries in population-based samples with the SOGS are not methodologically justified, since this questionnaire was developed as a *screening tool* and is therefore designed to increase sensitivity by minimizing the likelihood of false negatives: the objective of the SOGS is to identify potential “cases” of problem gamblers, under the assumption that further clinical assessment (through confirmatory-diagnostic tools such as clinical interviews) will resolve the misclassifications and eliminate the false alarms [32].

The fourth criticism of the SOGS is related to the structure. This tool has been routinely used in its original one-factor structure, but this organization has commonly been confirmed in small samples of clinical patients seeking treatment for GD. Alternative solutions incorporating more factors may achieve a more suitable fit with regard to the multiple qualitative dimensions underlying the gambling profile (in population-based and clinical settings). Recent research carried out in a large cross-sectional random sample from Finland (*n* = 4,484 individuals aged 15–74 years) found that a two-correlated bifactor model was adequate for the population-based sample (with two non-overlapping dimensions covering “gambling impact on self primarily” and “gambling impact on others also”), as well as a global dimension measuring gambling problem severity, and confirmed that the SOGS items were adequately aligned with the current DSM-5 criteria for GD [28]. However, these promising results regarding the multi-dimensionality of the SOGS have not been replicated and tested in other independent samples, or in studies analyzing data exclusively from older individuals.

## Objectives

Access to adequate tools for identifying gambling behaviors in elders is of the utmost importance. The purposes of this study are: a) to assess the presence of problematic and disordered gambling in older age, b) to test the adequacy of the bifactor structure for the SOGS [reported in the study of Salonen and colleagues [28]], and c) to obtain empirical evidence of the psychometrical properties of this questionnaire. These objectives are contrasted in a heterogeneous sample of over-50s including a population-based group recruited from the general population and a clinical group recruited from a Pathological Gambling Outpatient Unit.

## Materials and methods

### Participants

The data analysed in this study correspond to a research project developed at the Pathological Gambling Outpatient Unit at University Hospital of Bellvitge, designed to examine gambling habits in older age individuals and to explore the mechanisms underlying gambling severity.

Two samples were considered in the present study: participants recruited from the general population (“population-based sample”; *n* = 361) and patients recruited from the Pathological Gambling Outpatient Unit (“clinical sample”; *n* = 47). All 408 participants were recruited between November 2016 and February 2018. Inclusion criteria in the study were age 50+ years and a sufficient level of education and cognitive capacity to complete the self-report measures. The lower bound for classifying older adults was selected in 50 years based on the substantially variations in literature (usually from age 50+ to 70+ years). Exclusion criteria were the presence of an organic mental disorder, intellectual disability, neurodegenerative disorder (such as Parkinson's disease) or active psychotic disorder.

The population-based sample (*n* = 361) was recruited from the Podiatric and Dentistry clinics of the University of Barcelona, situated on the same campus as Bellvitge University Hospital, so as to guarantee that the two data sources had the same geographical origin. A non-probability sampling technique was used: all the subjects aged 50+ years-old who arrived to these two units during the same recruitment period than the clinical patients were invited to participate. The mean chronological age in this sample was 73.8 years (SD = 8.4) and the sex distribution was n = 135 men (37.4%) versus n = 226 women (62.6%). Most participants in this sample were born in Spain (*n* = 344, 95.3%), were married (*n* = 223, 61.8%) or widowed (*n* = 110, 30.5%), and reported primary level of education or lower (*n* = 309, 85.6%), and were retired (*n* = 354, 98.1%).

All the participants in the clinical sample (*n* = 47) met DSM-5 criteria for GD. The mean age in this sample was 70.0 years (SD = 5.6), and *n* = 37 (78.7%) were men and *n* = 10 (21.3%) were women. Most were married (*n* = 29, 61.7%) or widowed (*n* = 11, 23.4%), reported primary level of education or lower (*n* = 41, 87.2%), and were in retired (*n* = 44, 93.6%).

### Measures

*South Oaks Gambling Severity Screen (SOGS)* [24]. This is a 20-item instrument developed for measuring signs and symptoms of problem gambling and negative consequences over the last year. A total score obtained as the sum of the items is usually defined as a measure of gambling severity, with a score of 4 or more indicating problem gambling. This study used the Spanish validation of the scale, which achieved very good psychometrical results in the adaptation study (test-retest reliability R = 0.98, internal consistency α = 0.94 and convergent validity R = 0.92) [33].

#### Diagnostic Questionnaire for Pathological Gambling (according to DSM criteria) [20]

This is a self-report paper-and-pencil questionnaire with 19 items coded in a binary scale (yes-no), used for diagnosing GD according to the DSM-IV-TR [34]. This measurement instrument was used in this study as the reference gold standard for testing the SOGS screen [other previous studies had also used this tool as an external measure to assess the convergent validity of the SOGS [20,31,35–37]]. The DSM-IV measure was adapted to measure DSM-5 diagnostic criteria for Gambling Disorder [1] by removing the illegal acts criterion (resulting in nine criteria) and using a cutoff score of 4 to diagnose GD, rather than 5 as in DSM-IV-TR. Several measures for GD are allowed, based on the DSM-5 taxonomy: the presence/absence of each DSM criterion, the presence/absence of GD diagnosis, a dimensional measure of gambling severity (total number of DSM criteria, obtained as the sum of the individual criteria), and GD severity grouped in four levels [non-problem gambling (0 criteria), problem gambling (for 1–3 criteria), moderate GD (4–5 criteria), mild GD (6–7 criteria) and severe-GD (8–9 criteria)]. This questionnaire has demonstrated very good to excellent psychometrical properties [38]: internal consistency (Cronbach's alpha between α = 0.87 to α = 0.98), temporal reliability (intraclass correlation IC = 0.71for 1-week test-retest), convergent validity with external measures of gambling severity (the Pearson’s correlation with the SOGS was R = 0.97), and discriminative accuracy for differentiating between clinical versus population-based samples (hit rate range from 0.90 to 0.99, sensitivity range from 0.88 to 0.98, and specificity range from 0.83 to 0.99). The Spanish adaptation of the questionnaire used in this study obtained satisfactory psychometrical properties: internal consistency with a Cronbach’s alpha equal to α = 0.95 for the combined sample, satisfactory convergent validity (moderate to large correlations with other measures of problem gambling), and high discriminative capacity (sensitivity = 0.92 and specificity = 0.99) [39]. The internal consistency for this scale in the study sample was excellent (α = 0.92).

#### Symptom checklist-revised (SCL-90-R) [40]

This is a 90-item self-report pencil-and-paper instrument developed to assess a broad range of psychological symptoms and problems. It is structured in nine primary dimensions (somatization, obsessive-compulsive, interpersonal sensitivity, depression, anxiety, hostility, phobic anxiety, paranoid ideation and psychoticism) and three global indices (global severity index, GSI, total positive symptoms, PST, and positive discomfort index, PSDI). This study used the Spanish adapted version (internal mean α = 0.75) [41]. The internal consistency in the sample of this study ranged from good (α = 0.70, for the psychotic ideation scale) to excellent (α = 0.96 for the global indexes).

#### Other variables

Additional information analysed here was obtained through a semi-structured clinical interview, which included socio-demographics (e.g., sex, education, civil status and employment status) and other gambling problem-related variables (age of onset and duration of the gambling behaviors and bets per gambling/episode). This interview also assessed the different gambling activities, which allow group gambling behavior in three broad categories: non-strategic gambling (including those games which involve little decision-making or skill, and therefore gamblers cannot influence the outcome: slot-machines, bingo and lotteries), strategic gambling (including games in which gamblers attempt to use their ability to predict the outcome: poker, sports/animal betting, craps, etc.), and both non-strategic plus strategic. This specific tool has been described elsewhere [42].

### Procedure

All procedures were carried in accordance with the Declaration of Helsinki. This research was approved by the Ethics Committee of the Bellvitge University Hospital (Ref: PR286/14). All subjects were informed about the study and all provided informed consent. The semi-structured interview was conducted by psychologists and psychiatrists with extensive experience of over more than 15 years in the assessment and treatment of problematic gambling. They also helped participants to complete the self-report questionnaires to guarantee the absence of missing data.

### Statistical analyses

Confirmatory Factor Analysis (CFA) in MPlus8 for Windows tested the bifactor and the three-dimensional bifactor structures obtained in the study by Salonen and colleagues [28] for the Spanish version of the SOGS in the older age sample of our study. CFA was used in this work to confirm (or reject) the hypothetical construct underlying the SOGS based on the previous analytic research, and therefore the supposed number of factors required in the data and which measured variables were related to which latent variable were defined a priori. CFA modeling was performed adjusting for the covariates participants’ sex and age, and adequate goodness-of-fit was considered based on the usual standardized indexes [43]: root mean square error of approximation RMSEA < .08, Bentler’s Comparative Fit Index CFI>0.90, Tucker-Lewis Index TLI>0.90, and standardized root mean square residual SRMR<0.10. Following the statistical procedure of Salonen and colleagues, items with very low endorsement measuring a similar component of the gambling behavior were grouped into a single item to avoid convergence problems and non-adequate fitting in the CFA; additionally, since a high correlation was found between the two factors in the CFA, suggesting the possibility of a reinterpretation of the model as a three-dimensional bifactor model [44], this solution was also tested considering all the item loads on a single general three-factor model (labeled “total” in this study) and on a model of the two uncorrelated factors F1 and F2. This latter model was the one selected as optimal in the study by Salonen et al. [28].

Receiver Operating Characteristics (ROC) analysis was used to assess the global accuracy of the SOGS as a screening test for identifying the presence of gambling severity. This methodology is used in epidemiology and other research areas to quantify the validity of the screening and diagnostic tests (usually questionnaires, but also other systems) for differentiating between the patient states (typically referred to as diseased and non-diseased). In this study, the area under the ROC curve (AUC) was estimated as a measure of the global accuracy-validity of the SOGS across all the cutoff points, compared with the external reference-gold standard based on the DSM-5 criteria: disordered gambling (DSM-5 diagnosis: present versus absent) and problem gambling (DSM-5 severity level: at least problem gambling versus absent gambling). ROC analysis was also used to select the best cutoff point for the SOGS. Since the optimal cutoff depends on the hypothetical prevalence of the disorders and on the costs/risks of false classifications [32], the analysis was performed considering different scenarios: a) for hypothetical prevalence rates inside the range of 5% to 40%, and b) for ratios defining the cost of a false negative compared to a false positive classification between 1 to 5. Based on the main objective of screening tools (i.e., to identify at-risk individuals, which implies that costs of false negative classifications are higher than false positive ones), the selection of the optimal cutoff in the study was considered to be twice the cost of a false negative compared to a false positive screening score. The validity-accuracy for the final cutoff selected as optimal for the SOGS was measured through the usual indexes: sensitivity (Se), specificity (Sp), false alarm rate (FAR, the complement of the specificity), negative predictive value (PV−), positive predictive value (PV+), false discovery rate (FDR, the complement of the PV+), and Cohen’s-kappa (κ-coefficient, which estimated the agreement between the SOGS screening score with the reference diagnosis state based on the DSM-5; moderate-medium effect size was considered for κ>0.40, high-large for κ>0.60 and excellent for κ>0.80) [45].

Comparison between the groups for the SOGS scores was based on chi-square tests (χ^2^) for categorical measures (Fisher-exact test was used when expected frequencies e_ij_<5) and T-TEST for quantitative measures. Effect size for the proportion and mean differences was estimated with Cohen’s-*d* coefficient (low-poor effect size was considered for |*d*|>0.20, moderate-medium for |*d*|>0.5 and large-high for |*d*|>0.8) [46].

The convergent-discriminative validity of the SOGS scores versus the total number of DSM-5 criteria for GD and the psychopathological state (SCL-90R scales) was estimated with partial correlation indexes adjusted by sex and age. Estimations were obtained for the whole sample, as well as stratified by the origin of the sample (population-based versus clinical). Effect size was considered moderate for |*R*|>0.24, good for |*R*|>0.30 and large for |*R*|>0.37 [47].

In the study, since statistical differences between the population-based and the clinical samples were found for sex distribution and chronological age [with a higher prevalence of men (χ^2^ = 29.1, *df* = 1; *p* < .001) and younger mean age (*t* = 301, *df* = 406; *p =* .002) in the clinical setting], the participants’ sex and age were included as covariates in the statistical analysis to avoid potential biases due to the confounding effect of these variables.

## Results

### Gambling profiles in the study

Most participants into the population-based subsample (*n* = 361) reported 0 DSM-5 criteria for GD (*n* = 327, 90.6%), while *n* = 29 participants (8.0%) were into the problematic gambling group with 1–3 DSM-5 criteria for GD. The number of patients within this group who achieved DSM-5 criteria for GD was *n* = 5 (1.4%): 1 participant was in the low GD (0.3%, with 4–5 criteria) and 4 in the mild GD (1.1%, with 6–7 criteria). The number of participants who reported no gambling activity within this sample was *n* = 128 (35.5%), while *n* = 166 (46.0%) reported preference for non-strategic games, *n* = 8 (2.2%) reported preference for strategic games and *n* = 59 (16.3%) indicated gambling to both non-strategic and strategic games. The most preferred game in this group was lotteries (*n* = 218, 60.4%), followed by pools (*n* = 50, 13.9%) and bingo (*n* = 13, 11.9%). The age of onset of the gambling activity was 37.6 years (SD = 16.0) and the mean duration of the gambling behavior was 37.0 years (SD = 16.5). Only one participant in this group (0.3%) reported the presence of cumulate debts due to the gambling activity.

Into the clinical subsample (*n* = 47), the number of patients into the low GD was *n* = 22 (46.8%, with 4–5 criteria), into the mild was *n* = 16 (34.0%, with 6–7 criteria), and *n* = 9 patients (19.1%) were in the severe GD with 8–9 criteria. The number of participants who reported preference for non-strategic games was *n* = 28 (59.6%), the strategic subtype included *n* = 2 (4.3%) participants, and *n* = 17 (36.2%) individuals indicated preference for both non-strategic and strategic games. The most preferred game in this group was lotteries (*n* = 36, 76.6%), followed by slot-machines (*n* = 32, 68.1%), pools (*n* = 15, 31.9%) and bingo (*n* = 14, 29.8%). The age of onset of the gambling activity was 32.2 years (SD = 15.8) and the mean duration of the gambling behaviors was 38.3 years (SD = 14.3). The number of participants of this group who reported the presence of cumulate debts due to the gambling activity was *n* = 17 (36.2%).

### Factor structure of the SOGS

Among the total participants in the study (*n* = 408), the SOGS items with the highest endorsement were item 4 “gamble more than intended” (19.4% of positive responses) and item 6 “feel guilty due to gambling” (13.7% of positive responses), while some of the items referring to borrowing money (items 12 to 20) achieved the lowest endorsement (item 18 “cashed in stocks”, item 19 “sold personal or family property”, and item 20 “borrowed on checking account” obtained 0.2%, 0.7% and 0.2% of positive responses). Following the procedure of Salonen and colleagues, questions 12–20 were grouped into a single item defined as the presence of borrowing money from any of the specific sources [28].

**Fig 1:**
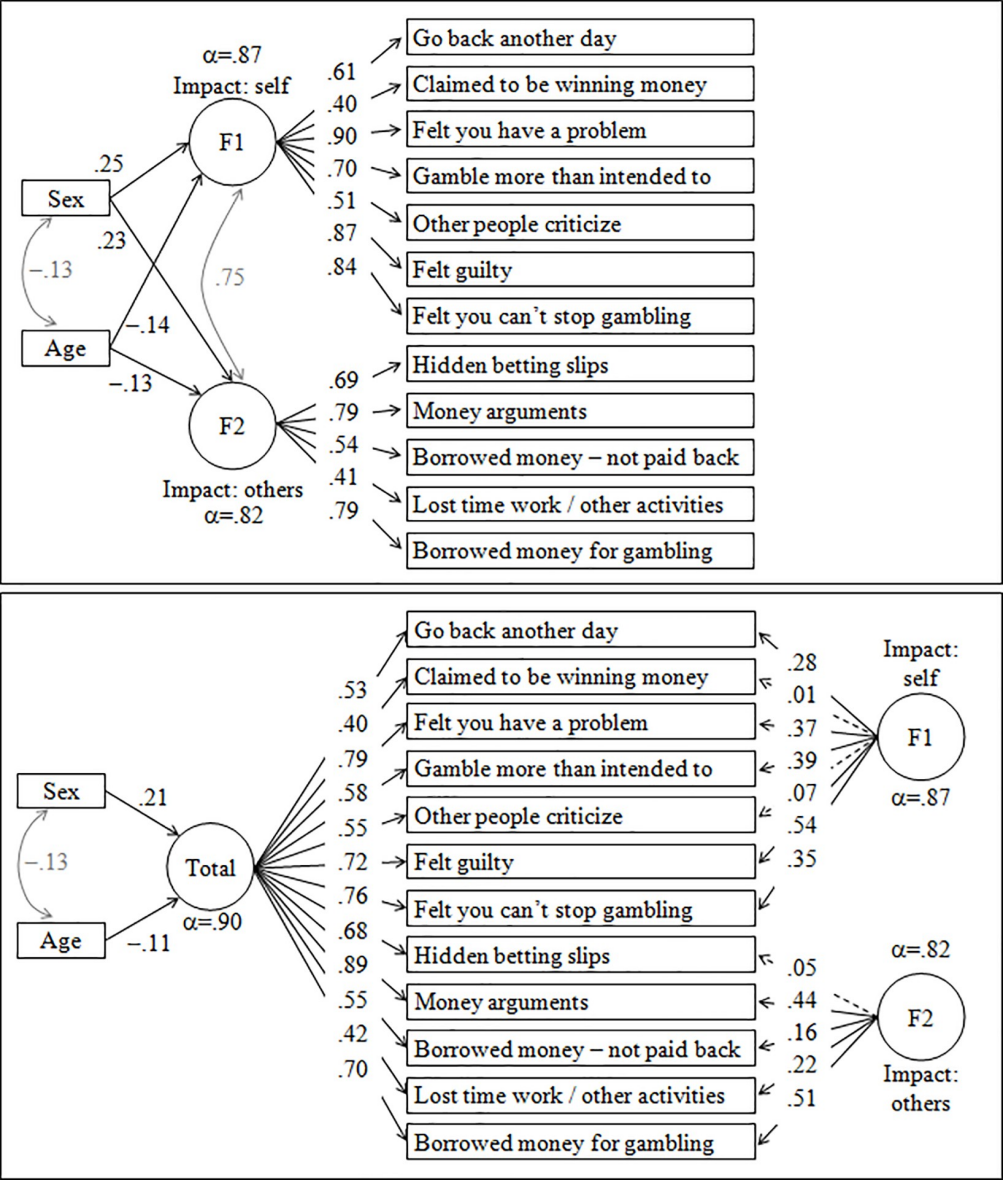
Path-diagrams with the standardized coefficients for three bifactor model in the study. Note. Continuous line: significant parameter. Dashed line: non-significant parameter. Grey color: covariance coefficients. α: Cronbach’s alpha.

The first panel in Fig 1 shows the path-diagram with the standardized coefficients of the CFA for the bifactor model in the study (the first block of S1 Table, supplementary material, contains the complete results for the model). Adjusted for sex and age, the bifactor model considering the two dimensions F1 “impact on the self primarily” and F2 “impact on others also” achieved an adequate fit: RMSEA = 0.039 (95% confidence interval: 0.024 to 0.053), CFI = 0.988, TLI = 0.981 and SRMR = 0.033. All the items obtained statistically significant standardized coefficients with high loadings. Very good internal consistency for both factors was also obtained: α = .87 and α = .82 for F1 and F2 respectively. The correlation between the two factors was high (*R* = 0.75).

The second panel in Fig 1 shows the path-diagram with the standardized coefficients for the bifactor model adding a general global latent factor, which was justified by the high correlation estimate between the two factors in the CFA displayed in Fig 1 (second block of S1 Table, supplementary material, contains the complete results for the model). Adjusted for sex and age, this new model achieved an adequate fit: RMSEA = 0.065 (95% confidence interval: 0.052 to 0.077), CFI = 0.965, TLI = 0.944 and SRMR = 0.038. Excellent internal consistency for the general total factor was obtained (α = .90). Many items in this model loaded onto the general factor (significant standardized coefficients) as well as onto the specific factors containing the primary impact area of gambling. The exceptions were item 2 “claimed to be winning money gambling but weren’t really”, item 5 “other people criticize the gambling activity” and item 8 “hidden betting slips”, which loaded significantly on the general factor but not on the F1 or F2 factors.

### Distribution of the SOGS scores

Table 1 shows the distribution of the SOGS scores (for each item and for the two dimensions) in the two samples. Comparison between the groups indicated that all the items achieved discriminative capacity for differentiating between the origin of the participants (population-based versus clinical), except for the items “borrowed money” “cashed in stocks” and “borrowed on checking account”.

**Table 1:** Distribution of the SOGS scores in the samples.

	Population-based (*n* = 361)			Clinic (*n* = 47)				
	*n*		*%*	*n*		*%*	*p*	*|d|*
01. Go back another day to win back money lost	10		2.8%	24		51.1%	< .001*	**1.30**^**†**^
02. Claimed to be winning money gambling	8		2.2%	10		21.3%	< .001*	**0.62**^**†**^
03. Felt having a problem with gambling	4		1.1%	43		91.5%	< .001*	**4.29**^**†**^
04. Gamble more than intended to	39		10.8%	40		85.1%	< .001*	**2.22**^**†**^
05.Other people criticize gambling	15		4.2%	21		44.7%	< .001*	**1.07**^**†**^
06. Felt guilty due to gambling	13		3.6%	43		91.5%	< .001*	**3.70**^**†**^
07. Felt can’t stop gambling	4		1.1%	40		85.1%	< .001*	**3.20**^**†**^
08. Hidden betting slips	14		3.9%	25		53.2%	< .001*	**1.30**^**†**^
09. Money arguments focused on gambling	6		1.7%	31		66.0%	< .001*	**1.85**^**†**^
10. Borrowed money and not paid it back	0		0.0%	11		23.4%	< .001*	**0.78**^**†**^
11. Lost time from work / other activities	2		0.6%	8		17.0%	< .001*	**0.61**^**†**^
12-20.Borrowed money for gambling from…	13		3.6%	31		66.0%	< .001*	**1.73**^**†**^
12. Household	10		2.8%	18		38.3%	< .001*	**0.98**^**†**^
13. Spouse/partner	2		0.6%	5		10.6%	< .001*	**0.51**^**†**^
14. Relatives	3		0.8%	3		6.4%	.022*	0.30
15. Banks	1		0.3%	16		34.0%	< .001*	**1.00**^**†**^
16. Credit cards	0		0.0%	18		38.3%	< .001*	**1.11**^**†**^
17. Loan sharks	0		0.0%	8		17.0%	< .001*	**0.64**^**†**^
18. Cashed in stocks	0		0.0%	1		2.1%	.115	0.21
19. Sold personal or family property	0		0.0%	3		6.4%	.001*	0.37
20. Checking account	0		0.0%	1		2.1%	.115	0.21
	*Mean*		*SD*	*Mean*		*SD*	*p*	*|d|*
F1: impact on self primarily	0.26		0.68	4.70		1.43	< .001*	**3.98**^**†**^
F2: impact on others also	0.11		0.41	3.15		2.48	< .001*	**1.71**^**†**^
Total score	0.36		0.97	7.85		3.28	< .001*	**3.09**^**†**^
Percentile estimates	F1	F2	Total	F1	F2	Total		
P_05_	0	0	0	2	0	3		
P_10_	0	0	0	3	1	5		
P_25_	0	0	0	4	1	6		
P_50_	0	0	0	5	2	7		
P_75_	0	0	0	6	5	10		
P_90_	1	0	1	6	7	13		
P_95_	1	1	2	7	8	14		

SD: standard deviation.

*Bold: significant comparison.

^†^Bold: effect size into the moderate-medium (|*d*|>0.50) to large-high range (|*d*|>0.80).

### Cut-off points for the SOGS

Compared with the reference measures for gambling based on the DSM-5 criteria, the SOGS total score obtained excellent accuracy for identifying the presence of disordered gambling (*AUC* = .97; 95% CI: 0.96 to 0.99) and also for classifying the level of problem gambling (*AUC* = .91; 95% CI: 0.86 to 0.96).

**Fig 2:**
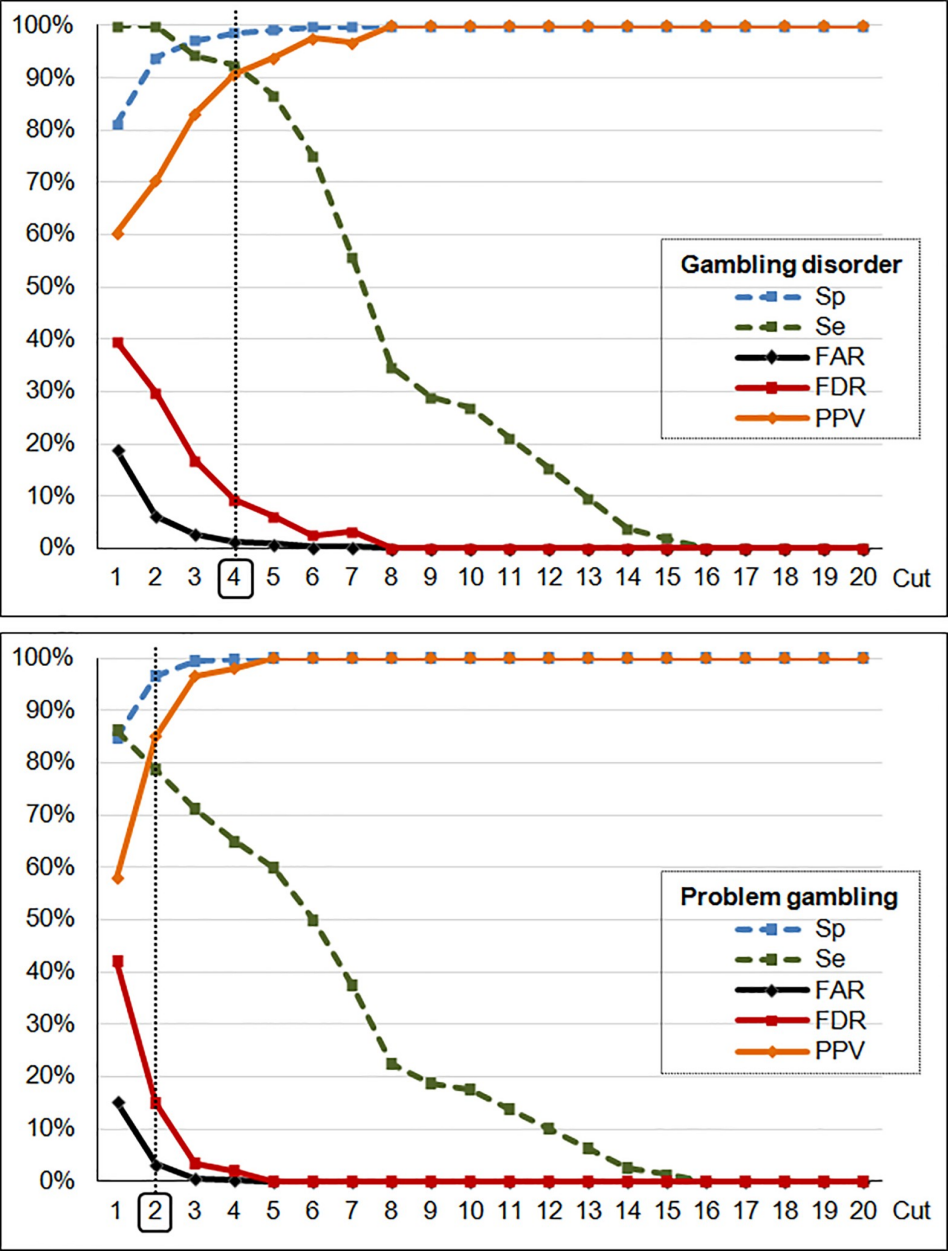
Screening capacity of the SOGS to identify gambling disorder and problematic gambling. Note. Sp: specificity. Se: sensitivity. FAR: false alarm rate. FDR: false discovery rate. PPV: positive predictive value.

Fig 2 displays the impact of the different potential SOGS cutoff points on the Se, Sp, FAR and PPV, with regard to the identification of the presence of GD and problem gambling in the sample (S2 Table, supplementary material, contains the complete results obtained in the ROC analysis for selecting the optimal cutoff point). In this study, defining the cost of a negative false classification as twice that of a positive false classification, the optimal cutoff point for the SOGS as a screening tool for identifying the presence of disordered gambling (GD present versus absent) was 4 (*Se* = 92.3%, *Sp* = 98.6%, FAR = 1.4%, PV+ = 90.6%, PV− = 98.9% and Cohen’s κ = 0.90), and the best cutoff point for identifying the problematic gambling was 2 (*Se* = 78.8%, *Sp* = 96.7%, FAR = 3.4%, PV+ = 85.1%, PV− = 94.9% and Cohen’s κ = 0.78).

### Association between the SOGS with external measures

Table 2 contains the partial correlations matrix (adjusted for sex and age) between the SOGS raw scores with the total number of DSM-5 criteria for GD and the global psychopathological state (SCL-90R scores). As a whole, considering the total participants in the study (*n* = 408), high relevant correlations were achieved between SOGS scores with all the external measures, except the phobic anxiety level and bets per gambling-episode. As expected, as higher the SOGS score as higher the gambling severity (measured through the number of DSM-5 criteria, the number of gambling activities and the amount of debts related with the gambling problems) and as worse the psychopathological state.

**Table 2:** Association between the SOGS with external measures: partial correlations (adjusted for the participants’ sex and age).

	Total (*n* = 408)			Population-based (*n* = 361)			Clinic (*n* = 47)		
*SOGS measures* →	F1	F2	Total	F1	F2	Total	F1	F2	Total
*Gambling measures*									
Number of DSM-5 criteria for GD	**.905**^**†**^	**.794**^**†**^	**.914**^**†**^	**.620**^**†**^	**.586**^**†**^	**.687**^**†**^	**.596**^**†**^	**.559**^**†**^	**.685**^**†**^
Number of gambling activities	**.451**^**†**^	**.384**^**†**^	**.450**^**†**^	**.350**^**†**^	**.331**^**†**^	**.388**^**†**^	.234	.197	**.252**^**†**^
Bets per gambling-episode (mean, euros)	**.242**^**†**^	.163	.220	.011	-.011	.003	.065	-.095	-.045
Bets per gambling-episode (max., euros)	**.332**^**†**^	.185	**.284**^**†**^	.069	-.029	.036	.080	-.132	-.067
Debts due to gambling activity	**.549**^**†**^	**.613**^**†**^	**.618**^**†**^	.053	**.365**^**†**^	.194	**.296**^**†**^	**.387**^**†**^	**.424**^**†**^
*Psychopathology*: *SCL-90R*	** **								
Somatization	**.267**^**†**^	.191	**.249**^**†**^	**.274**^**†**^	.157	**.260**^**†**^	**.290**^**†**^	.142	.233
Obsessive-compulsive	**.310**^**†**^	**.250**^**†**^	**.302**^**†**^	**.301**^**†**^	.170	**.284**^**†**^	.233	.232	**.278**^**†**^
Interpersonal sensitivity	**.358**^**†**^	**.373**^**†**^	**.390**^**†**^	**.308**^**†**^	.192	**.299**^**†**^	**.442**^**†**^	**.617**^**†**^	**.663**^**†**^
Depression	**.500**^**†**^	**.402**^**†**^	**.487**^**†**^	**.307**^**†**^	.165	**.286**^**†**^	**.305**^**†**^	.221	**.300**^**†**^
Anxiety	**.427**^**†**^	**.318**^**†**^	**.404**^**†**^	**.344**^**†**^	.169	**.314**^**†**^	**.377**^**†**^	.214	**.326**^**†**^
Hostility	**.301**^**†**^	**.311**^**†**^	**.327**^**†**^	**.342**^**†**^	.157	**.307**^**†**^	**.304**^**†**^	**.526**^**†**^	**.534**^**†**^
Phobic anxiety	.179	.136	.170	**.268**^**†**^	.135	**.246**^**†**^	.209	**.281**^**†**^	**.305**^**†**^
Paranoid ideation	**.284**^**†**^	**.255**^**†**^	**.290**^**†**^	**.296**^**†**^	.186	**.288**^**†**^	.117	**.306**^**†**^	**.285**^**†**^
Psychotic ideation	**.439**^**†**^	**.377**^**†**^	**.439**^**†**^	**.271**^**†**^	.114	**.240**^**†**^	**.297**^**†**^	**.312**^**†**^	**.367**^**†**^
GSI	**.442**^**†**^	**.366**^**†**^	**.436**^**†**^	**.374**^**†**^	.203	**.350**^**†**^	**.394**^**†**^	**.373**^**†**^	**.455**^**†**^
PST	**.373**^**†**^	**.293**^**†**^	**.360**^**†**^	**.359**^**†**^	.183	**.330**^**†**^	**.331**^**†**^	**.319**^**†**^	**.387**^**†**^
PSDI	**.317**^**†**^	**.243**^**†**^	**.303**^**†**^	.106	.073	.106	**.359**^**†**^	.114	**.241**^**†**^

F1: impact on self primarily. F2: impact on others also.

^†^Bold: effect size into the moderate-medium (|*R*|>.24) to large-high range (|R|>.37).

Stratified by the origin of the sample, diverse relevant correlation emerged into both groups showing also that as higher the SOGS score as higher the scores in the other measures of the gambling severity and higher psychopathological distress. The correlation pattern revealed in the two subsamples was however different: a) the coefficients tended to achieve higher effect size among the clinical individuals than in the population-based group; and b) within the population-based sample, the factor F2 “impact on others also” only achieved relevant correlations with the number of DSM-5 criteria for GD, the total number of gambling activities and the debts accumulated due to gambling, but no relevant association emerged between this factor with the psychopathological state.

## Discussion

A number of screening and diagnostic self-report tests have been developed and validated for problem gambling among youths and young- to middle-age adults, but no empirical evidence is available about the validity of these tools for identifying problematic and disordered gambling in older individuals. This study aimed to explore the gambling profile in older age and to assess the psychometrical properties of the SOGS in a population-based sample and a clinical sample of patients who sought treatment for gambling-related problems. The CFA confirmed the bifactor structure (*impact on the self primarily area / impact on others also*), with an additional global general dimension of the gambling problem severity. Additional analyses obtained good discriminant and convergent validity compared with external measures of gambling (including the DSM-5 criteria) and with the psychopathological state, thus highlighting the adequate psychometrical properties of the SOGS for elders.

The gambling profiles obtained in the study showed that the age of onset and the duration of the gambling behavior were quite similar within the clinical and the community subsamples. In addition, non-strategic games were the most preferred form of gambling regardless of the origin of the sample. These results are coherent with previous studies examining preferred style of gambling, which observed that older age individuals tend to select games which involve little decision-making with the aim to escape from emotional distress, while high rates of arousal-seeking behavior should be the reasons for younger age gamblers preferring strategic gambling forms [12,48–51]. The age-related cognitive dysfunctions have also been related with the non-strategic gambling preference in older age individuals, who usually exhibit worse performance in the decision-making processes [52,53]. On the other hand, the higher prevalence of strategic gambling within the clinical sample (compared to the population based sample) is also particularly interesting, since these games at older ages (alone or concurrent with non-strategic gambling) have be identified as a warning sign of higher risk of problematic gambling or disordered gambling [54].

Regarding the frequency of the GD, the prevalence of this disorder in the population-based sample was 1.4% (*n* = 5 participants met clinical criteria for this disorder) while 8.0% of individuals were into the problematic gambling group (between 1–3 criteria). These point estimates within the community sample were within the range of the cross-sectional worldwide estimates published in epidemiological systematic reviews: between 0.3% to 10.4% in adults over the age of 50 [3] and between 0.01% to 10.6% in samples of adults over 60 [2]. The relatively low prevalence in our study must be interpreted with caution, since some studies alert that aging adults could perceive and/or recognize the negative consequences of the gambling activities only when these adverse impacts have occurred [55].

Regarding the heterogeneity of the gambling profile in elders, few studies have focused on this topic. Based on the evidence available regarding the evolution of gambling activity over the life span, two groups were identified in clinical samples of GD seniors (lifetime gamblers with low levels of impairment, versus current problem gambling patterns with higher impairment) [56], and three main groups in the population-based sample (elders without history of gambling-related impairment and without current GD, subjects without a history of gambling-related impairment who increased their gambling during older age and became problem gamblers, and seniors with long-standing gambling behaviors who maintained the gambling habits already acquired and presented chronic GD) [57]. As regards the motivations for gambling and psychological variables, three main pathways to late-life problem gambling have been reported in clinical settings [6]: a grief pathway associated with unresolved losses, a pathway explained by the habituation to gambling, and a dormant pathway defined by high levels of impulsivity. These results, however, do not explain the potential differences in gambling profile related to the behaviors measured with the SOGS, and confirm the need for future research in this area.

An important contribution of this work is to present empirical evidence of the hierarchical nature of the SOGS in the elderly age: the two single factors illustrate the multidimensional nature of the gambling behavior and the general global dimension supports the existence of a superordinate gambling factor explaining the covariation among the individual responses. These findings are relevant for research and clinical purposes. Firstly, all the items achieved significant factor loadings and made stronger contribution to the general factor than to their specific factor, which is the empirical justification for the scoring of the SOGS in a single dimensional measure based on the sum of all the items. This total score is, precisely, the measure usually employed by clinicians and researchers to place individuals along a continuum of gambling severity, based on the outcomes in the domains assessed in the questionnaire. Secondly, the scores obtained in the two specific factors (measuring the primary impact areas) provide information about the clinical manifestation of the gambling problem, which is also useful to classify phenotypes and to determine the negative impact of gambling (both for the patients themselves and for their significant others) [58]. Thirdly, the results regarding the dimensionality of the SOGS are particularly relevant considering that this questionnaire was originally developed according to the operational definition of pathological gambling based on the DSM-III criteria, and no modifications have been introduced in this classical tool in the last 30 years. Since many prevalence and etiological studies in clinical and population-based samples continue to use the SOGS today to screen and diagnose GD (for a large age range, from young adults to the elderly), it is essential to report psychometrical evidence of the adequate structure of gambling problems with this tool compared to reference gold standard measures based on the current DSM-5 version.

In this study, SOGS items 12–20 valuing “borrowing money from different sources” obtained low endorsement. The study by Salonen and colleagues also found low endorsement for this group of items (as in the present study, they generated a single measure in the CFA analyses) [28], as well as other psychometric studies [27,31,35]. The particularly low endorsement of these items in the population-based samples could be considered arguing strongly against the use of the SOGS as a real 20-item measure for gambling practices, especially in the general population. The rationale for including a large number of items measuring borrowing money in the development of the original version of this tool was that these behaviors represent relatively mild forms of problem gambling [24,30]. However, epidemiological research in recent years suggests that these items are in fact related to the more severe forms of disordered gambling. In fact, the assessment of borrowing money in the SOGS is related strongly with the presence of illegal acts related to gambling activity (such as forgery, fraud, theft, or embezzlement to finance gambling) [59]. Although borrowing money from any source to gamble or to pay off the debts accumulated is not *per se* an illegal act, gamblers are very unlikely to pay the money back: they usually argue that they initially intended to return the money later, but the delay in returning it may in practice be illegal [60–62].

Illegal behaviors related to gambling were removed as a diagnostic criterion in the last version of the DSM (DSM-5) [1], due to their low prevalence in clinical and population-based samples (they were infrequently endorsed in the absence of other criteria) and their low discriminative capacity for identifying the presence of GD [62,63]. But although the diagnostic capacity of illegal behavior is limited in a taxonomy such as the DSM, this criterion has proved to have important implications in research and clinical settings, which justify its retention in a measurement instrument such as the SOGS. Specifically, illegal acts have been associated both with the severity of GD and with increases in patients’ impairment and distress [64,65]. Having several items measuring the possible presence of illegality activity rather than just one may seem excessive in a screening tool such as the SOGS, but it would be advisable to maintain them, for two main reasons: a) some individuals may be unaware that their activity is illegal, because they had planned to return the money later (for example, when they had recovered it), because they do not perceive the act to be illegal in the absence of legal rulings, or even because they are unaware that certain behaviors are forbidden by law; or b) some individuals may answer “no” to the direct question of “illegal acts” during the assessment (especially in the initial phases) because they fear the potentially adverse implications of admitting these behaviors (in fact, it is preferable to assess this criterion using alternative verification items). As an example, in our study item 10 “borrowed money from someone and not paid them back” was not endorsed by any individuals in the population-based sample, while 13 subjects (3.6%) acknowledged borrowing money to support their activities (items 12 to 20); in the clinical sample, item 10 was endorsed by 11 patients (23.4%), while 31 patients (66.0%) endorsed items 12–20 assessing borrowing money with the aim to gamble or finance gambling-related debts. It should also be in mind that some previous studies have found that specific items in the SOGS are of special relevance since they assess different levels of risk gambling. Concretely, the study of Holtgraves obtained that borrowing money was more characteristic of high risk gamblers compared to other items (such as chasing losses, felling guilty or betting more than could afford) which are more characteristics of low risk [66]. The study of Miller and colleagues carried out in a large nationally representative sample also found that borrowing money registered low endorsement (1.7%) and the lowest loading in factorial analysis, but it revealed the high discriminative capacity to identify high levels of gambling severity and it was the only item to display both statistically and clinically meaningful differential item functioning between game type subgroups [67]. The low endorsement could even be the result that the wording in the SOGS result ambiguous for some individuals who could be interpreting the item in some different ways. If this was the case, the item could be improved by providing examples of what borrowing constitutes.

The items with the highest endorsement differed according to the sample. In the population-based group the most highly endorsed questions were “gamble more than intended” and “other people criticize your gambling”, and in the clinical group the highest endorsement rates were for “feel you have a problem with gambling” and “feel guilty due to the gambling behavior”. This result suggests differences in the experience and perception of potential gambling problems in population-based and clinical groups, as well as the existence of different phenotypes depending on the origin of the sample. Regarding the awareness of problem gambling, a recent study observed that older people only perceive harm and impairment once the most adverse consequences of gambling have already occurred [55], which may explain why in our study the items measuring “feeling you have a problem” and “feeling guilty” received high endorsement only in the clinical sample. This result is particularly relevant to epidemiological research estimating the prevalence of GD in the general population, since it may explain the low rates recorded in elders in early observational studies. It also highlights the risk that aging individuals who indeed present problem gambling may not receive professional treatment.

In this study, the best cutoff for identifying the presence of GD was 4, with excellent accuracy (*Se* = 92.3%, *Sp* = 98.6%, *FAR* = 1.4% and *FDR* = 9.4%). But the optimal cutoff for identifying patients with at least high risk of problematic or disordered gambling was 2, which obtained poorer validity (*Se* = 78.8%, *Sp* = 96.7%, *FAR* = 0.6% and *FDR* = 14.9%). These results reinforce the validity of the SOGS as a dimensional measure of gambling severity in older individuals, and highlight the problems with the use of the cutoff of 2 to screen for potential problem gamblers in the general population: the low sensitivity means that around 20% of true cases would not be adequately identified, and that around 15% of the positive screen scores in the SOGS would be non-real cases of problematic gamblers. These results need to be carefully considered taking into account the different uses of this measurement tool according to the origin of the samples (community versus clinical settings). The use of the SOGS as a screening tool in population-based samples has the potential to be a cost effective method for identifying high risk subjects (for example with early-stage disease or low severity of the gambling activity). But these instruments have the disadvantage of relatively high risk of false discovery, precisely because their predictive value depend in part of the technical parameters of the instruments (including sensitivity and specificity) but also on the prevalence of the disorders in the populations. Therefore, the results obtained for the SOGS in this study should not be considered poor for screening problematic gambling and disordered gambling among older age: this developmental stage constitute a vulnerable group and the consequences of false negatives should be more serious than false positives (untreated problems may cause more significant impairment than the costs and consequences of false positive diagnoses). Regarding the clinical settings, it should be appropriate to use the SOGS as a dimensional assessment of the gambling severity (rather than a screening/diagnostic tool), that is, a measurement of the level of the gambling behavior within a continuum of the symptom profile and its consequences.

Finally, the relevant correlations obtained in the study between the SOGS score with other measures of the gambling severity and the psychopathological state provides empirical evidence about the convergent validity of the SOGS and its factorial structure. Pathological gambling usually has a wide range of adverse consequences on individuals, including emotional and mental health problems. Previous studies also found associations between gambling severity and poorer psychological health status in older age [12,15,56,68,69], but studies on the temporal relationships are limited. While our results reinforce the hypothesis that the psychopathological state could contribute to the onset and/or the progress of gambling problems in older adults, and the supposition that gambling behavior could be a powerful risk factor for the onset and/or increase of the psychopathological distress. Older patients with mood and anxiety disorders could resort to gambling as a means of escaping from the negative states. But depression and stress observed in older pathological gamblers could not be primary to underlying gambling symptoms, but constituted a secondary reaction to the negative consequence of pathological gambling such as family breakdown, isolation or financial problems.

### Limitations

One limitation of the study is the analysis of data obtained through self-reports. These measures are commonly used in the psychiatric area (in both clinical and research settings). Two advantages of these assessment tools are that they can be performed relatively easy and that they can be made in private allowing in some cases more truthful responses. However, collecting data through self-reports has limitations and biases, such as the lack of introspective ability (individuals may not be able to assess themselves accurately) or the difficult for interpretation of the questions (different meanings could be suggested by the wording of the items).

Other limitation is the low sample size of the clinical group, which decreased the statistical analysis power (increasing the likelihood of Type II error) and avoided to obtain a more accurate/precise picture of the gambling phenotype. Additionally, since factor analysis has been generally considered a technique for large sample sizes, it could be supposed that the low number of GD patients influenced the dimensionality of the SOGS. However, the current factor analysis literature suggest that there are no minimum recommended ratio between the number of subjects and the number of variables, mainly because the coefficients estimates are dependent on several characteristics beyond the sample size (such as the level of correlation among factors or the number of variables which achieve adequate factor loading to define each factor) [70,71].

The sampling selection in this study could also affect the results generated with the factor analysis: although the CFA achieved an adequate fit in this work, there is no guarantee that other potential structures would obtain goodness-of-fit in larger samples containing more heterogeneous profiles for disordered gambling, or selecting a large sample representative of a well defined population. However, it must be considered that factor analysis requires sampling strategies which allow the selection of participants who are likely to present the complete range of values for the construct of interest, since homogeneous groups restrict variance and reduce factor loadings. In this sense, current simulation studies prove that for factor analysis covering a diverse range of scores for the construct under study takes precedence over selecting individuals from a identified population [72,73].

Regarding the population-based sample in this study, it constituted an availability-convenience group (individuals were recruited without a probability sampling procedure), and this may affect the generalizability of the results because the potential vulnerability to latent selection bias. It must be argued, however, that non-probability sampling is usually used in clinical research due its simplicity and its helpfulness for pilot studies and for hypothesis generation.

### Strengths

The main strength of the study is the inclusion of individuals recruited from different settings, since the data analysed correspond to a population-based sample and a clinical sample of patients who sought treatment for disordered gambling. This heterogeneity in the sampling procedure allowed us to obtain relevant information about the psychometrical properties of the SOGS, as well as about the potential structure of the most common problematic gambling behaviors in different areas.

### Conclusion and implications

The high prevalence of gambling activity in older individuals, and the increase in problematic gambling among elders expected in the coming years, call for the design of reliable and valid tools for accurately diagnosing the presence of GD in clinical settings and for screening the warning signs of the disorder in at-risk population-based groups. The results provide empirical evidence that the psychometric properties of the three-dimension bifactor structure of the SOGS are adequate for screening problem gambling at older ages. Our results also reinforce the validity of the dimensionality of the SOGS (originally developed according to the operational definition of gambling in the DSM-III taxonomy) as a screening measure for identifying the presence of GD and the severity of problem gambling according to the DSM-5. Future research should examine the utility of brief versions of the SOGS for effectively screening elders at high risk of problem gambling. The use of this approach has obtained interesting results in studies based on the item response theory analysis, such as the research by Strong and colleagues [36] which used a Rash model for the analysis of data from a population-based sample and a large clinical sample to reduce the original 20-item version to a 6-item version covering the DSM-IV criteria, and achieved excellent psychometrical properties by assessing the gambling severity levels. Access to brief reliable screening tools for problem gambling behaviors would be particularly useful in settings such as primary care, which elders attend on a routine basis.

## Supporting information

S1 Table Confirmatory factorial analysis (standardized coefficients). (DOCX)

S2 Table ROC analysis to select the optimal cutoff point for the SOGS. (DOCX)
