# Supplementary material for: Presence of problematic and disordered gambling in older age and validation of the South Oaks Gambling Scale
Source: PLoS One. 2020 May 19;15(5):e0233222. doi: 10.1371/journal.pone.0233222 (PMC7237015; doi:10.1371/journal.pone.0233222)
Supplement: S1 Table — (DOCX) [file pone.0233222.s001.docx]

*Table S1 (supplementary)*

| *Confirmatory factorial analysis (standardized coefficients)*Bifactor model | | Coeff | SE | T-stat | *p* | 95%CI coeff. | |
| --- | --- | --- | --- | --- | --- | --- | --- |
| Structural | F1: sex | 0.254 | 0.048 | 5.25 | <0.001 | 0.159 | 0.349 |
|  | F1: age | -0.139 | 0.050 | -2.81 | 0.005 | -0.236 | -0.042 |
|  | F2: sex | 0.226 | 0.050 | 4.49 | <0.001 | 0.127 | 0.325 |
|  | F2: age | -0.126 | 0.051 | -2.47 | 0.014 | -0.226 | -0.026 |
| Measurement | F1: Go back another day to win money | 0.614 | 0.033 | 18.42 | <0.001 | 0.549 | 0.680 |
|  | F1: Claimed to be winning money gambling | 0.404 | 0.045 | 9.00 | <0.001 | 0.316 | 0.492 |
|  | F1: Felt you have a problem with gambling | 0.900 | 0.015 | 60.98 | <0.001 | 0.871 | 0.929 |
|  | F1: Gamble more than intended to | 0.699 | 0.029 | 24.36 | <0.001 | 0.643 | 0.755 |
|  | F1: Other people criticize gambling | 0.512 | 0.039 | 13.20 | <0.001 | 0.436 | 0.588 |
|  | F1: Felt guilty due to gambling | 0.866 | 0.016 | 52.77 | <0.001 | 0.834 | 0.898 |
|  | F1: Felt you can’t stop gambling | 0.842 | 0.020 | 42.09 | <0.001 | 0.803 | 0.881 |
|  | F2: Hidden betting slips | 0.688 | 0.032 | 21.48 | <0.001 | 0.625 | 0.750 |
|  | F2: Money arguments focused on gambling | 0.792 | 0.030 | 26.34 | <0.001 | 0.733 | 0.851 |
|  | F2: Borrowed money and not paid back | 0.543 | 0.041 | 13.30 | <0.001 | 0.463 | 0.623 |
|  | F2: Lost time from work / other activities | 0.415 | 0.047 | 8.82 | <0.001 | 0.323 | 0.507 |
|  | F2: Borrowed money for gambling from… | 0.794 | 0.028 | 28.74 | <0.001 | 0.740 | 0.848 |
| Three-dimensional bifactor model | | Coeff | SE | T-stat | *p* | 95%CI coeff. | |
| Structural | Total: sex | 0.210 | 0.049 | 4.31 | <0.001 | 0.115 | 0.306 |
|  | Total: age | -0.108 | 0.050 | -2.19 | 0.029 | -0.205 | -0.011 |
| Measurement | Total: Go back another day to win money | 0.531 | 0.038 | 13.80 | <0.001 | 0.456 | 0.606 |
|  | Total: Claimed to be winning money Total | 0.400 | 0.046 | 8.74 | <0.001 | 0.310 | 0.490 |
|  | Total: Felt you have a problem with gambling | 0.786 | 0.023 | 33.61 | <0.001 | 0.740 | 0.832 |
|  | Total: Gamble more than intended to | 0.584 | 0.036 | 16.16 | <0.001 | 0.513 | 0.655 |
|  | Total: Other people criticize gambling | 0.546 | 0.037 | 14.80 | <0.001 | 0.474 | 0.618 |
|  | Total: Felt guilty due to gambling | 0.718 | 0.030 | 24.20 | <0.001 | 0.659 | 0.776 |
|  | Total: Felt you can’t stop gambling | 0.755 | 0.027 | 27.98 | <0.001 | 0.702 | 0.808 |
|  | Total: Hidden betting slips | 0.684 | 0.030 | 22.42 | <0.001 | 0.624 | 0.743 |
|  | Total: Money arguments focused on gambling | 0.897 | 0.023 | 38.58 | <0.001 | 0.851 | 0.942 |
|  | Total: Borrowed money and not paid back | 0.548 | 0.039 | 14.14 | <0.001 | 0.472 | 0.624 |
|  | Total: Lost time from work / other activities | 0.423 | 0.048 | 8.89 | <0.001 | 0.330 | 0.516 |
|  | Total: Borrowed money for gambling from… | 0.705 | 0.035 | 20.30 | <0.001 | 0.637 | 0.773 |
| Measurement | F1: Go back another day to win money | 0.284 | 0.051 | 5.54 | <0.001 | 0.184 | 0.385 |
|  | F1: Claimed to be winning money gambling | 0.000 | 0.068 | 0.00 | 0.997 | -0.132 | 0.133 |
|  | F1: Felt you have a problem with gambling | 0.366 | 0.040 | 9.23 | <0.001 | 0.288 | 0.444 |
|  | F1: Gamble more than intended to | 0.387 | 0.046 | 8.50 | <0.001 | 0.297 | 0.476 |
|  | F1: Other people criticize gambling | 0.070 | 0.054 | 1.31 | 0.192 | -0.035 | 0.176 |
|  | F1: Felt guilty due to gambling | 0.540 | 0.045 | 12.03 | <0.001 | 0.452 | 0.628 |
|  | F1: Felt you can’t stop gambling | 0.354 | 0.060 | 5.88 | <0.001 | 0.236 | 0.472 |
|  | F2: Hidden betting slips | 0.049 | 0.048 | 1.02 | 0.307 | -0.045 | 0.144 |
|  | F2: Money arguments focused on gambling | 0.437 | 0.100 | 4.38 | <0.001 | 0.241 | 0.632 |
|  | F2: Borrowed money and not paid back | 0.161 | 0.052 | 3.08 | 0.002 | 0.059 | 0.264 |
|  | F2: Lost time from work / other activities | 0.224 | 0.052 | 4.32 | <0.001 | 0.122 | 0.326 |
|  | F2: Borrowed money for gambling from… | 0.515 | 0.100 | 5.17 | <0.001 | 0.320 | 0.710 |

*Note.* SE: standard error: T-stat: T-statistic.
