# Supplementary material for: Presence of problematic and disordered gambling in older age and validation of the South Oaks Gambling Scale
Source: PLoS One. 2020 May 19;15(5):e0233222. doi: 10.1371/journal.pone.0233222 (PMC7237015; doi:10.1371/journal.pone.0233222)
Supplement: S2 Table — (DOCX) [file pone.0233222.s002.docx]

*Table S2 (supplementary)*

*ROC analysis to select the optimal cutoff point for the SOGS*

| Hypothet. | Reference → | Identify GD | | | | | Identify problematic gambling | | | | |
| --- | --- | --- | --- | --- | --- | --- | --- | --- | --- | --- | --- |
| Prevalence | FNC → | 1 | 2 | 3 | 4 | 5 | 1 | 2 | 3 | 4 | 5 |
| 5% | Cutoff | 5 | 4 | 4 | 4 | 4 | 5 | 3 | 3 | 3 | 3 |
|  | Se (%) | 86.5 | 92.3 | 92.3 | 92.3 | 92.3 | 60.0 | 71.3 | 71.3 | 71.3 | 71.3 |
|  | Sp (%) | 99.2 | 98.6 | 98.6 | 98.6 | 98.6 | 100 | 99.4 | 99.4 | 99.4 | 99.4 |
| 10% | Cutoff | 4 | 4 | 4 | 4 | 4 | 3 | 3 | 3 | 2 | 2 |
|  | Se (%) | 92.3 | 92.3 | 92.3 | 92.3 | 92.3 | 71.3 | 71.3 | 71.3 | 78.8 | 78.8 |
|  | Sp (%) | 98.6 | 98.6 | 98.6 | 98.6 | 98.6 | 99.4 | 99.4 | 99.4 | 96.6 | 96.6 |
| 20% | Cutoff | 4 | 4 | 2 | 2 | 2 | 3 | 2 | 2 | 2 | 2 |
|  | Se (%) | 92.3 | 92.3 | 100 | 100 | 100 | 71.3 | 78.8 | 78.8 | 78.8 | 78.8 |
|  | Sp (%) | 98.6 | 98.6 | 93.8 | 93.8 | 93.8 | 99.4 | 96.6 | 96.6 | 96.6 | 96.6 |
| 30% | Cutoff | 4 | 2 | 2 | 2 | 2 | 2 | 2 | 2 | 1 | 1 |
|  | Se (%) | 92.3 | 100 | 100 | 100 | 100 | 78.8 | 78.8 | 78.8 | 86.3 | 86.3 |
|  | Sp (%) | 98.6 | 93.8 | 93.8 | 93.8 | 93.8 | 96.6 | 96.6 | 96.6 | 84.8 | 84.8 |
| 40% | Cutoff | 2 | 2 | 2 | 2 | 2 | 2 | 2 | 1 | 1 | 1 |
|  | Se (%) | 100 | 100 | 100 | 100 | 100 | 78.8 | 78.8 | 86.30 | 86.30 | 86.30 |
|  | Sp (%) | 93.8 | 93.8 | 93.8 | 93.8 | 93.8 | 96.6 | 96.6 | 84.8 | 84.8 | 84.8 |
| Optimal cutoff | | 4 | | | | | 2 | | | | |
| Prevalence (sample) | | 12.70% | | | | | 19.60% | | | | |
| FNC / FPC | | 2 / 1 | | | | | 2 / 1 | | | | |
| Sensitivity | | 92.31% (95% CI: 81.83 to 96.97) | | | | | 78.75% (95% CI: 68.58 to 86.29) | | | | |
| Specificity | | 98.60% (95% CI: 96.75 to 99.40) | | | | | 96.65% (95% CI: 94.10 to 98.12) | | | | |
| Positive predictive value | | 90.57% (95% CI: 64.55 to 98.06) | | | | | 85.14% (95% CI: 62.90 to 95.08) | | | | |
| Negative predictive value | | 98.87% (95% CI: 93.81 to 99.80) | | | | | 94.91% (95% CI: 87.83 to 97.97) | | | | |
| Kappa | | 0.902 (95% CI: 0.838 to 0.965) | | | | | 0.776 (95% CI: 0.697 to 0.855) | | | | |

*Note.* FNC: false negative cost. FPC: false positive cost.
